# Sinonasal immunoglobin G4—related disease: case report and review

**DOI:** 10.1002/ccr3.5095

**Published:** 2021-12-04

**Authors:** Caleb P. Wilson, Benjamin P. Brownlee, Edward T. El Rassi, Kibwei A. McKinney

**Affiliations:** ^1^ Department of Otolaryngology University of Oklahoma Health Sciences Center Oklahoma City Oklahoma USA

**Keywords:** chronic sinusitis, IgG4‐related disease, sinonasal disease

## Abstract

Immunoglobin G4—related disease (IgG4‐RD) is a chronic fibro‐inflammatory condition that presents as a single or multiple tumefactive lesions affecting virtually any organ system. Here we report a case of recurrent sinonasal IgG4‐RD and review the literature of this evolving entity.

## 1 INTRODUCTION

Immunoglobin G4‐related disease (IgG4‐RD) is a chronic fibro‐inflammatory disease that presents as one or multiple tumefactive lesions affecting virtually any organ system. It was first described in 2001 by Hamano et al.^1^ in patients with autoimmune pancreatitis and elevated serum IgG4 levels. In 2011, an international consensus on the diagnosis of IgG4‐RD was developed, which requires histologic evidence of IgG4 positivity in addition to other hallmark features including lymphoplasmacytic infiltration, storiform fibrosis, obliterative phlebitis, and mild to moderate tissue eosinophilia.^2^ The exact etiology of IgG4‐RD is poorly understood, but recent studies have shown a clear role of type 2 helper T cells and T regulatory cells. IgG4 itself seems to be a protective response to the inappropriate immune activation, rather than pathologic.^3^

While first described in the pancreas, manifestations of the disease have been described throughout the body, including the lacrimal glands, submandibular glands (Kuttner tumor), orbit, lymph nodes, thyroid, biliary tract, liver, kidney, retroperitoneum, lungs and even aorta. Mulholland et al.^4^ demonstrated that the condition shows a predilection for the head and neck, with the orbit or salivary glands being the most frequently involved. Although the head and neck region is the second most common location of involvement for IgG4‐RD, the paranasal sinuses are rarely affected, and the first sinonasal case was described in 2009 by Ishida et al.^5^ In 2015, Song et al.^6^ published the first sinonasal‐specific IgG4‐RD literature review in which it was described as an emerging entity. At that time, only 8 cases had been described in the literature that met the current diagnostic criteria, but since that review, many other cases have been reported.^2^ This report will add to the existing data on sinonasal IgG4‐RD with the present case, which describes the longest‐known course of this disease in a single patient, spanning over 22 years. A comprehensive review of the literature on this topic will then be provided. Finally, strategies for the early recognition and diagnosis of IgG4‐RD will be discussed and the efficacy of sinus‐specific treatment modalities will be reviewed.

## 2 METHODS

A comprehensive literature review using PubMed and Web of Science was performed through February 2021 following Preferred Reporting Items for Systematic Reviews and Meta‐Analyses (PRISMA) guidelines. Articles related to IgG4‐related disease with sinonasal involvement were identified using the search terms “IgG4‐related disease,” “sinonasal IgG4,” and “sinus IgG4.” Upon removing duplicates, titles and abstracts were screened for relevant articles. English, full‐text case reports and series were included. Non‐English, abstract‐only text, and review articles were excluded. Studies that did not meet histopathological diagnostic criteria for IgG4‐related disease as described by Deshpande et al.^2^ were further excluded. Ultimately, 20 references meeting the selection criteria were included.^5^, ^6^, ^7^, ^8^, ^9^, ^10^, ^11^, ^12^, ^13^, ^14^, ^15^, ^16^, ^17^, ^18^, ^19^, ^20^, ^21^, ^22^, ^23^, ^24^ The epidemiology, presentation, imaging characteristics, locations of involvement, IgG4 serum and histopathologic levels, treatments rendered, and follow‐up of all 31 cases (when available) were extracted and summarized.

## 3 CASE REPORT

This is the case of a 72‐year‐old male patient whose disease course began with the development of a spontaneous cerebrospinal fluid (CSF) leak in 1999. His early clinical course, from the onset of symptoms up until 2012, was described by Hu et. al.^25^ in the *Annals of Allergy*, *Asthma*, and *Immunology* in 2013, which this manuscript will briefly recount. It is the goal of this paper to present his disease course following Rituximab infusion, as well as the long‐term follow‐up between 2013 and 2021. This patient's disease initially manifested with multiple systemic features, including pulmonary emboli, xerostomia, and intermittent double vision; however, the early stage of disease is not the focus of this paper and is, therefore not extensively discussed.

In 1999, at the age of 50 years, the patient presented with CSF rhinorrhea and was incidentally noted to have left ethmoid sinusitis on computed tomography (CT). He underwent a craniotomy for repair, and the surgeon noted that that the dura had a 2 mm defect in the area of the left cribriform plate. The area of infection in the left ethmoid was not addressed. His repair was completed without complication and he remained asymptomatic for over a decade.

He presented to another otolaryngologist in 2012 following a 12‐month history of recurrent acute sinusitis. Imaging at that time, including CT and magnetic resonance imaging (MRI) revealed opacification of the left‐sided paranasal sinuses, hyperostotic bone, and a contrast‐enhancing lesion of the left ethmoid cavity that was concerning for malignancy. Endoscopic sinus surgery was performed shortly thereafter, revealing diffuse mucosal inflammation without evidence of a discrete mass lesion. Multiple biopsy specimens were sent intraoperatively. The final pathology revealed a lymphoplasmacytic infiltrate with areas of fibrosis in a storiform pattern, as well as plasma cells that stained positive for IgG4. His IgG4/IgG ratio was >80% in the ethmoid specimen, >40% in tissue from the maxillary sinus, and serum IgG4 was within normal limits (88 mg/dl; normal <121 mg/dl). He was initially diagnosed with inflammatory pseudotumor; however, the report was appended several weeks later, and the diagnosis of IgG4‐RD was subsequently made.

Shortly after diagnosis, he was referred to an IgG4‐RD specialist in Boston, MA and underwent two consecutive Rituximab infusions. One month after the infusions, his IgG4 level was reduced to 55 mg/dl. The patient noted symptomatic relief, specifically a reduction in sinus pressure and pain, and in‐office nasal endoscopy confirmed a marked improvement in the degree mucosal inflammation. He remained asymptomatic for over a year but his symptoms returned, once again manifesting with left‐sided sinus pressure, pain, and purulent rhinorrhea.

Conservative treatment with Budesonide irrigation and culture‐directed antibiotics failed to adequately manage his symptoms. A post‐treatment CT scan revealed opacification of residual left‐sided ethmoid air cells, hyperostotic bone and mucosal thickening within the left maxillary sinus. He underwent revision endoscopic sinus surgery, including left maxillary antrostomy, total ethmoidectomy, and frontal sinus exploration, which was complicated by a CSF leak from the left lateral lamella of the cribiform plate. This was repaired with a free mucosal graft from the left middle turbinate. His surgical pathology showed an IgG4/IgG ratio that was >70%.

Over the following year, his chronic rhinosinusitis (CRS) symptoms were successfully managed with nasal saline irrigations and intermittent use of oral steroids and culture‐directed antibiotics for disease exacerbations. The frequency of infections and the number of antibiotics prescribed increased over the following year, with worsening polypoid edema in the left maxillary sinus on follow‐up endoscopy. A third Rituximab infusion was performed in 2016, and at that time his IgG4 level was 45 mg/dl. Unlike his prior infusions, his symptoms did not improve as dramatically, and he had four additional sinus infections requiring antibiotics over the ensuing 12 months. A left maxillary mega‐antrostomy was performed in October 2016, and again the pathologic evaluation of the tissue supported the diagnosis of IgG4‐RD. Following surgery, he was again started on Budesonide irrigations which he continued daily for over a year, after which he transitioned to irrigations with buffered saline alone. Over the following years, he has remained mostly asymptomatic and only required intermittent culture‐directed antibiotics for sinusitis exacerbations. At his most recent clinic appointment in December of 2020, his SinoNasal Outcome Test‐22 (SNOT‐22) score was 3, and he had not experienced a sinus infection for over a year.

## 4 DISCUSSION

IgG4‐RD is a rare fibro‐inflammatory condition characterized by tumefactive lesions that can arise in multiple different sites. First described in 2001 by Hamano et al.,^1^ this disease process has several areas that remain to be understood, including how to recognize it. IgG4‐RD is difficult to diagnose because it can mimic a variety of autoimmune and neoplastic diseases, and it also has non‐specific radiologic features. A patient with primarily or exclusively sinonasal involvement may be uniquely challenging to diagnose, especially if they present with classic features of CRS.

### 4.1 Location

While it is common for IgG4‐RD to involve multiple different organ systems at presentation, it seems that sinonasal IgG4‐RD is often limited to local disease or only head and neck involvement. Of the 31 cases in this review, 14 (45%) had extra‐sinonasal involvement, most of which involved the salivary glands or orbit. Only 2 of these cases involved organs outside of the head and neck, and the present case is the third. The most common sinonasal structures involved were the maxillary sinus (61%), followed by the ethmoid sinus (29%), nasal cavity/septum (26%), sphenoid sinus (19%), and two cases involving the frontal sinus (6%). The sinonasal locations of involvement and other organs involved are shown in Table 1. Because sinonasal IgG4‐RD can often present exclusively within the sinuses, it is important for rhinologists and general otolaryngologists to know when it is appropriate to consider this entity among the differential diagnosis.

**TABLE 1:** Clinical, radiologic, and immunohistologic features of cases from literature review

	Age/Gender	Sinonasal location	Imaging characteristics	Serum IgG4 (g/L)	IgG4+ plasma cells/HPF	Immunochemistry IgG4+/IgG+ ratio	Other organs involved
Ishida et al. (2009)^5^	73/M	Nasal septum Maxillary	Mass without bony destruction	0.63	“Diffuse”	>70%	Parotid gland
Ikeda et al. (2010)^7^	50/F	Ethmoid Maxillary	Mass without bony destruction	2.58	39	77%	Pleura
Sasaki et al. (2011)^8^	71/M	Maxillary Nasal cavity	Mass + bony destruction	1.14	“Numerous”	N/a	
Alt et al. (2012)^9^	38/F	Sphenoid	Mass + bony destruction	0.2	>150	N/a	
Lindau et al. (2012)^10^	69/M	Ethmoid Maxillary Sphenoid	Mass + bony destruction	0.74	>30	N/a	Orbit
Prabhu et al. (2014)^11^	15/F	Maxillary Nasal septum	Mass + bony destruction	2.06	N/a	50%	
Prabhu et al. (2014)^11^	15/F	Maxillary Ethmoid Sphenoid Nasal cavity	Mass + bony destruc tion	5.79	>30	50%	
Morris et al. (2014)^12^	34/M	Nasal cavity	Mass without bony destruction	2.3	>50	>40%	
Kurien et al. (2015)^13^	21/M	Maxillary	Mass + bony destruction	1.97	70	>70%	Pterygoid plates Orbital floor
Kojima et al. (2015)^14^	67/M	Maxillary Sphenoid Frontal	N/a	2.43	N/a	>40%	
Song et al. (2015)^6^	72/M	Maxillary Ethmoid	Mass without bony destruction	0.945	>50	N/a	Orbit
Inoue et al. (2015)^15^	70/M	Sphenoid	Fluid in sinuses	1.98	34	59%	
Chen et al. (2016)^16^	36/M	Ethmoid Nasal cavity	Mass without bony destruction	1.62	>100	N/a	
Vandjelovic et al. (2016)^17^	46/M	Ethmoid Frontal	Mass + bony destruction	N/a	N/a	>50%	
Gontarz et al. (2016)^18^	30/M	Ethmoid Maxillary	“Thick soft tissues”	3.35	75	80%	Upper alveolar mucosa Cervical lymph nodes
Bashyam et al. (2018)^19^	71/F	Maxillary Nasal cavity	Mass without bony destruction	1.6	“Marked increase”	70%	
Chowsilpa et al. (2019)^20^	19/F	Maxillary Nasal cavity	“Soft tissue swelling” + bony destruction	1.1	10 to 30	N/a	Middle ear
Ueno et al. (2020)^21^	69/F	“Paranasal”	Gland swelling + fluid in sinuses	5.24	“many”	>40%	Lacrimal glands Parotid gland Submandibular gland Gall bladder
Ueno et al. (2020)^21^	69/F	“Paranasal”	Gland swelling + fluid in sinuses	11.8	N/a	>50%	Lacrimal glands Parotid gland Submandibular gland
Ueno et al. (2020)^21^	64/F	“Paranasal”	Fluid in sinuses	13.7	N/a	80%	Lacrimal glands Parotid gland Submandibular gland
Ueno et al. (2020)^21^	41/F	“Paranasal”	Fluid in sinuses	1.62	N/a	N/a	Lacrimal glands Parotid gland Submandibular gland
Ueno et al. (2020)^21^	39/F	“Paranasal”	Gland swelling + fluid in sinuses	7.21	N/a	>40%	Lacrimal glands Parotid gland Submandibular gland
Jurkov et al. (2020)^22^	77/M	“Paranasal”	“Intraorbital enhancement”	1.659	N/a	N/a	Orbit
Kouwenberg et al. (2020)^23^	48/M	Maxillary	Mass + bony destruction	0.56	135	40%	Maxillary alveolar process
Kaur et al. (2021)^24^	48/M	Ethmoid Maxillary	Soft tissue enhancement + bony destruction	1.82	23	N/a	
Kaur et al. (2021)^24^	29/F	Sphenoid	Enhancing lesion in sphenoid	2.1	50	N/a	
Kaur et al. (2021)^24^	13/M	Maxillary	Mass + bony destruction	1.9	23	N/a	
Kaur et al. (2021)^24^	38/F	Maxillary	Soft tissue enhancement + bony destruction	2.29	105	N/a	
Kaur et al. (2021)^24^	35/F	Maxillary	Mass + bony destruction	2.24	22	N/a	
Kaur et al. (2021)^24^	19/M	Maxillary Ethmoid	Soft tissue enhancement	N/a	43	N/a	
Kaur et al. (2021)^24^	32/F	Maxillary	N/a	N/a	36	N/a	
Our Case	63/M	Maxillary Ethmoid	Soft tissue enhancement	0.88	Diffuse	80%	

### 4.2 Epidemiology

IgG4‐RD has been shown to be most prevalent in the older male population, with a reported male‐to‐female ratio of 1.6–4:1.^26^, ^27^, ^28^ The most recent review of sinonasal IgG4‐RD by Song et al.^6^ was consistent with this gender distribution. However, this review demonstrates a nearly 1:1 ratio between males and females (16:15, or 53% male). Interestingly, Mulholland et al.^4^ also reported a ratio of nearly 1:1 for all head and neck involvement. Additionally, the average age of diagnosis for IgG4‐RD has been reported to be within the 6th decade of life, but the review of cases with sinonasal involvement presented here demonstrates a younger average age of presentation(46 years), with a few cases diagnosed in patients as young as 13, as summarized in Table 2.

**TABLE 2:** Basic patient demographics

Total number of patients	31
Average age (years)	46
Percent male	53

### 4.3 Presenting symptoms

Common presenting symptoms of sinonasal IgG4‐RD from this article's review include nasal obstruction/congestion (48%), facial swelling (26%), headache (19%), and epistaxis or blood‐tinged rhinorrhea (16%). While only five other cases (16%) presented a multiple‐year history of chronic rhinosinusitis like this patient, IgG4‐RD might be an important diagnostic consideration in patients with intractable CRS, especially when imaging is suggestive of the disease. Additionally, a few other cases described recurrent facial pain, congestion, and nasal discharge that were consistent with CRS in whom this diagnosis was not made. These presenting symptoms are, therefore, important to consider when making the diagnosis of sinonasal IgG4‐RD. Presenting symptoms are listed in Table 3.

**TABLE 3:** Presenting symptoms (*n *= 31)

Symptom	Percentage
Nasal obstruction/congestion	48
Facial swelling	26
Headache	19
Epistaxis/bloody rhinorrhea	16
CRS	16

For the patient in the present case, sinonasal endoscopy was performed at each of the clinic appointments during the nine years he was managed at this institution. The modified Lund‐Kennedy (mLK) endoscopic scoring system is a standardized method of quantifying the visual appearance of the sinuses on nasal endoscopy.^29^ The reliability of this system is improved by excluding the subscores of scarring and crusting, and correlates more closely to patient‐reported outcome measures such as the SNOT‐22, in both non‐operated and postoperative patients.^29^ Table 4 denotes the date of assessment, LK score for the left and right nasal cavity, and the isolated bacteria and culture‐directed antibiotics that were used to treat the patient.

**TABLE 4:** Modified Lund‐Kennedy Score and treatment modality

Date	Left mLK Score	Right mLK Score	Culture	Antibiotics
12/15/2020	0	0		
12/17/2019	2	1		
12/14/2018	0	0		
12/15/2017	2	0	*Staphylococcus aureus*	No antibiotics
6/9/2017	0	0		
2/24/2017	0	0		
1/20/2017	3	0		
12/16/2016	1	0	*Pseudomonas aeruginosa* and *Staphylococcus aureus*	Cefixime 400 mg × 2 weeks and Bactrim 800 mg × 2 weeks
11/30/2016	2	0		
11/4/2016	2	0		
9/28/2016	5	0	*Pseudomonas aeruginosa* and *Staphylococcus aureus*	Cirpofloxacin 500 mg × 3 weeks and Bactrim 800 mg × 3 weeks
3/25/2016	4	4	*Pseudomonas aeruginosa* and *Staphylococcus aureus*	Ciprofloxacin 500 mg × 3 weeks and Bactrim 800 mg × 4 weeks
11/25/2015	2	0	*Pseudomonas aeruginosa*	Ciprofloxacin 500 mg × 3 weeks
1/23/2015	2	0	*Staphylococcus aureus*	Bactrim 800 mg × 4 weeks
9/26/2014	1	0	*Staphylococcus aureus*	Doxycycline 100 mg × 4 weeks
5/23/2014	2	0	*Hemophilus influenzae* and *Staphylococcus aureus*	Bactrim 800 mg × 6 weeks
2/21/2014	2	0	*Streptococcus pneumoniae*	Clindamycin 300 mg × 3 weeks
1/17/2014	1	0		
1/3/2014	2	1		
12/27/2013	3	0		
10/16/2013	2	0	*Staphylococcus aureus*	Bactrim 800 mg × 4 weeks

### 4.4 Imaging characteristics

The majority of cases demonstrated thickening, attenuation, or enhancement of the soft tissue in the sinuses with or without a discreet mass. However, Ueno et al.^21^ describe 5 cases where imaging revealed only fluid retention in the involved sinuses. Importantly, bony destruction or erosion was reported in the imaging description of 13 (45%) of these cases. It is possible that additional cases had this feature on imaging but did not report it. Because bony erosion or tissue destruction might lead a clinician to suspect malignancy, it is important to rule this out histologically. In the present case, there was soft tissue enhancement without a discrete mass or bony destruction.

### 4.5 Immunochemistry and histopathology

Definitive international pathology consensus guidelines for diagnosis were formally described in 2012 by Deshpande et al.^2^ Because serum IgG4 levels are neither sensitive nor specific for IgG4‐RD, histopathology is required to confirm the diagnosis. The hallmark histologic findings for IgG4‐RD include lymphoplasmacytic infiltration, storiform fibrosis, obliterative phlebitis, and mild to moderate tissue eosinophilia. While the exact findings and numbers of IgG4‐positive cells vary depending on the tissue affected, the diagnosis of IgG4‐RD is based primarily on IgG4‐positive to IgG‐containing cell ratio and the number of IgG4 positive cells per high powered field. A ratio of IgG4 to total IgG >40% or IgG4 positive cells per high‐power field >10 is considered to be diagnostic of IgG4‐RD. While serum IgG4 levels are not diagnostic alone, a level >135 mg/dl is also suggestive of IgG4‐RD and may correlate with the severity of disease.^2^, ^30^

Elevated serum levels of IgG4 are usually reported in 70%–80% of all patients with IgG4‐RD,^31^, ^32^ although the results of a study by Carruthers et al.^30^ found that the sensitivity for detecting disease with a serum IgG4 levels >135 mg/dl might actually be as high as 90%. The present review of sinonasal IgG4‐RD is consistent with these findings, with elevated serum levels in 75% of cases (*N* = 28). All of the cases that reported a value of IgG4+ plasma cells/HPF >10 (19 cases), with most values >30 (79%). Several others reported “diffuse” or “many” IgG4+ plasma cells instead of numeric values. All of the cases that reported IgG4+ cells/total IgG‐containing cells ratio demonstrated a ratio >40% (16 cases). Laboratory and histologic summary from the present review is detailed in Table 5.

**TABLE 5:** Laboratory and histologic findings

	Average values	Number of patients
Serum IgG4 (mg/dl)	294.9	25
IgG4+ cells/HPF	56.9	18
IgG4+ cells/IgG‐containing cells	59%	19

### 4.6 Treatment

There are many approaches to treatment of IgG4‐RD which usually involve some combination of surgical resection of affected tissues, systemic glucocorticoids, steroid‐sparing immunosuppressive drugs and biological agents. Prednisone monotherapy at a dose of 0.6 mg/kg/day and tapered over the period of 3–6 months is currently considered the first‐line therapy.^26^

In a recent study, the recurrence rate for IgG4‐RD was reported to be 46% after initial treatment with steroids.^26^ However, this review of sinonasal disease demonstrated that, of the 14 patients who initially underwent steroid monotherapy, only 1 had recurrent disease (7%). In that case, the addition of rituximab led to clinical improvement. Of the 2 patients who only underwent surgical resection and debulking for whom follow‐up was reported, both developed disease recurrence. However, Inoue et al.^15^ described one patient with sinusitis and no notable mass lesions who underwent sinus surgery alone, and showed no evidence of recurrence after a year. Of the 5 patients who had both surgery and steroid treatment, 1 had recurrent disease (20%) which improved after the addition of rituximab. While one case presented by Kaur et al.^24^ reported no recurrence with the use of rituximab alone, this was not the experience of the patient presented here. While the patient did have initial improvement with his initial two infusions of rituximab in 2012, his disease recurred a year later and persisted despite a third infusion of rituximab and intermittent oral steroids. However, after additional surgical resection of the affected maxillary sinus with mega‐antrostomy and steroid rinses, the patient has been symptom free and without evidence of clinical recurrence for 5 years. Treatment and follow‐up outcomes from the cases in this article's review are described in Table 6.

**TABLE 6:** Treatment and follow‐up

Initial treatment received (*N *= 23)	Total number	Remission achieved	% Remission achieved	Cases with progression or recurrence	Treatment added to achieve remission
Surgical management alone	3	1	33	Ishida et al.	N/a
				Alt et al.	1st Steroid, later required 2nd Steroid + Rituximab
Combined surgical and corticosteroid management	5	4	80	Lindau et al.	2nd Steroid + Rituximab
Corticosteroid alone	14	13	93	Prabhu et al.	Rituximab
Mycophenolate alone	1	0	0	Kaur et al.	Rituximab, later required surgery
Rituximab alone	1	1	100		

## 5 CONCLUSION

While a greater sample size is required to draw definitive conclusions, the findings presented here, which include the most comprehensive review of literature to date, suggest that the general otolaryngologist or rhinologist should have a high index of suspicion for this disease process when considering patients who present with new‐onset symptoms of chronic sinusitis, particularly middle‐aged adults without a longstanding history of sinonasal complaints or patients with refractory disease despite appropriate surgical intervention. When sinonasal IgG4‐RD is considered, the authors of this article recommend diagnostic workup that includes CT sinuses without contrast, serum IgG4, and biopsy of the involved sinus tissue, if feasible. Although the utility of positron emission tomography‐computed tomography (PET‐CT) in the diagnosis of IgG4‐RD has been supported, sinonasal IgG4‐RD is often limited to the head and neck, and therefore, it should not be used for the initial diagnostic workup. PET‐CT can be considered once the diagnosis has been confirmed in order to identify other organ system involvement or to monitor treatment response.^33^

Additionally, these findings suggest that steroid monotherapy is usually sufficient to prevent recurrence in sinonasal IgG4‐RD. Although surgery may be indicated to improve symptoms, caution may be warranted, since it does not seem to reduce the likelihood of recurrence. The findings of this case and literature review also confirm that rituximab is an important consideration with recurrent disease after steroid monotherapy. A summary of diagnostic workup recommendations by the authors of this report can be found in Table 7.

**TABLE 7:** Diagnostic workup

CT Sinus w/o contrast
Serum IgG4
Tissue biopsy
PET‐CT—only after definitive diagnosis

## CONFLICTS OF INTEREST

This research received no specific grant from any funding agency in the public, commercial or not‐for‐profit sectors. There are no competing interests with regard to this project.

## AUTHOR CONTRIBUTION

Caleb P. Wilson, Ben Brownlee, Kibwei A. McKinney, and Edward El Rassi each contributed meaningfully to data collection, manuscript writing, and final review.

## CONSENT

Patient consent was obtained for inclusion in this case report. No identifying details have been disclosed.

## Data Availability

Data sharing not applicable to this article as no datasets were generated or analyzed during the current study.
